# Association between obstructive sleep apnea and venous thromboembolism recurrence: results from a French cohort

**DOI:** 10.1186/s12959-021-00358-8

**Published:** 2022-01-04

**Authors:** Olivier Nepveu, Charles Orione, Cécile Tromeur, Alexandre Fauché, Cecile L’heveder, Marie Guegan, Catherine Lemarié, David Jimenez, Christophe Leroyer, Karine Lacut, Francis Couturaud, Raphael Le Mao

**Affiliations:** 1 Groupe d’Etude de la Thrombose de Bretagne Occidentale, UMR 1304 , INSERM, , Département de médecine vasculaire, interne et pneumologie, Centre hospitalo-universitaire de Brest, Université de Bretagne Occidentale, CHRU de Brest, 29609 Brest, Cedex France; 2 Centre d’Investigation Clinique INSERM 1412, Brest, France; 3 Respiratory Department, Hospital Ramón y Cajal and Instituto Ramón y Cajal de Investigación Sanitaria (IRYCIS), CIBER de Enfermedades Respiratorias (CIBERES), Instituto de Salud Carlos III, Madrid, Spain

## Abstract

**Background:**

Growing evidence suggests the relationship between obstructive sleep apnea (OSA) and venous thromboembolism (VTE). Few studies focused on VTE recurrence risk associated with OSA after anticoagulation cessation.

**Methods:**

In a prospective cohort study, patients with documented VTE, were followed for an indefinite length of time and VTE recurrence were documented and adjudicated. The primary outcome was recurrent VTE after anticoagulation discontinuation. Secondary outcomes included all-cause mortality and the clinical presentation of VTE. Univariable and multivariable analyses were performed to identify risk factors for recurrence and mortality.

**Results:**

Among the 2109 patients with documented VTE included, 74 patients had moderate to severe OSA diagnosis confirmed by home sleep test or polysomnography. During a median follow-up of 4.8 (interquartile range 2.5–8.0) years recurrent VTE occurred in 252 patients (9 with OSA and 243 without OSA). The recurrence risk in the univariable and multivariable analysis was not increased in patients with OSA, regardless of the time of diagnosis (before or after index VTE or pooled). VTE phenotype was significantly more often PE with or without associated deep vein thrombosis in the first event and recurrence for OSA patients compared to non-OSA patients. The risk of death was not increased in the OSA population compared to non-OSA patients in multivariable analysis.

**Conclusions:**

In patients with OSA and VTE, the risk of all-cause mortality and VTE recurrence after anticoagulation discontinuation was not increased compared to non-OSA patients.

**Supplementary Information:**

The online version contains supplementary material available at 10.1186/s12959-021-00358-8.

## Introduction

Venous thromboembolism (VTE), including pulmonary embolism (PE) and deep vein thrombosis (DVT), is a major health issue. PE is the most common cardiovascular cause of death after myocardial infarction and stroke [1] with an annual incidence of 0.69/1000 [2, 3]. The most frequent complication is VTE recurrence (10% at 1 year, 25% at 5 years for unprovoked VTE), with a mortality rate of 4% [4]. Identifying transient or permanent risks factor of VTE is the cornerstone of medical care of patients [5].

Obstructive sleep apnea (OSA), characterized by periodic narrowing and obstruction of the pharyngeal airway during sleep [6], is also a common disease (3 to 10% of the general population) [7–9], probably underestimated [10] and associated with the onset of cardiovascular and metabolic comorbidities [11–17]. VTE and OSA have some risk factors in common such as age, obesity and immobility [18].

There is a growing evidence suggesting that OSA is a risk factor of VTE [19, 20]. Several physiological studies have explored and demonstrated the effect of OSA on coagulation and suggested that OSA induced a prothrombotic state, an oxidative stress, a chronic inflammation and vasoconstriction, increased coagulation factors, and altered fibrinolysis [20]. Moreover, obesity is frequently associated with OSA (up to 45% of OSA diagnosis in obese patients) [21] and is also a risk factor of VTE [22–24]. OSA prevalence is higher in patients with previous VTE compared to the general population [25]. Concerning the association between OSA and VTE recurrence, Alonso-Fernandez et al.*,* in a case-control study including 107 patients with PE and 102 patients without VTE, showed that OSA was more frequent in patients with PE and for every 10-unit rise in apnea-hypopnea index (AHI), the PE risk increased by 45% [26]. Though this study suggested an increased risk of VTE recurrence in OSA patients, prospective studies showed some conflicting results regarding VTE recurrence risk [27–29].

In the present study, we aimed to assess the association between OSA and recurrent VTE after a first event of VTE and anticoagulation discontinuation. We analyzed, as well, the correlation between AHI and nocturnal desaturation with thrombotic risk and the impact of OSA on mortality.

## Methods

### Study design

Consecutive patients with objectively diagnosed VTE in four French Hospital Centers were included in a prospective multicenter cohort study [30–32]. All cases had an unlimited follow-up with the annual clinical information collection. All VTE cases that occurred between May 2000, and June 2019 were included in the present report.

### Patient selection

Consecutive patients hospitalized or referred to Hospital outpatient VTE clinics for documented VTE (e.g., an isolated symptomatic DVT or symptomatic PE associated or not to DVT) who discontinued anticoagulation after VTE and during follow-up were potentially eligible. Patients were then indexed at time of anticoagulation discontinuation.

### Index VTE diagnosis

The diagnosis of VTE was performed using objective, standardized and validated criteria (45,46). Symptomatic DVT was confirmed in case of a non-compression of deep veins of the legs using real-time B mode ultrasound. Symptomatic PE was confirmed if there was: (i) a high clinical probability and a high-probability ventilation-perfusion lung scan according to the PIOPED criteria, or (ii) a proximal DVT showed by ultrasonography in a patient with symptoms of PE, or (iii) a positive computed tomography pulmonary angiography (CTPA) showing a central filling defect outlined by contrast material or complete occlusion in a segmental or more proximal pulmonary artery. VTE was classified,according to recent french guidelines, as provoked in the presence of at least one of the following transient or persistent major risk factors: surgery or immobilization in the past three months, pregnancy or post-partum in the past three months, cancer, administration of an estrogen-containing pill, hormone replacement therapy, pregnancy or the post-partum period within the previous 3 months [33] . VTE was considered as unprovoked in the absence of all these risk factors.

### OSA identification

In this study, we focused on moderate to severe OSA in order to select a homogeneous population of patients supposed to be at higher risk of VTE events. OSA was therefore defined in high probability pretest for sleep apnea, when AHI was greater than 15 or patient with OSA diagnosis requiring dedicated device (i.e., continuous positive airway pressure or non-invasive ventilation). To identify all patients with respiratory diseases and OSA, research by ICD 10 code and keywords was conducted in the Brest University Hospital database and crossed with the cohort study database. The use or initiation of CPAP or NIV in the 12-months following the diagnosis was also collected but without collecting initiation date. The diagnosis of OSA was confirmed for each patient based on medical records, seeking pulmonary functional tests, home sleep tests, and polysomnography results by one or two experienced physician(s). In the case of missing HST and polysomnography (PSG) results in our database, missing data were collected directly from the patient’s pulmonologist records. As well, OSA diagnosis was confirmed two times. In the analysis, we distinguished two groups of OSA patients: “OSA-history” was defined as those with OSA diagnosed prior to index VTE event and “OSA-occurrence” as those with OSA diagnosed after the index VTE event.

### Follow-up

All patients were prospectively followed up to five years, with an annual collection of clinical, biochemical, and morphological data. Interviews were conducted initially and during follow-up using the same standardized questionnaire. After the first 3 to 6 months of anticoagulant treatment, patients were followed systematically annually through a dedicated visit or a phone call. Investigations were made in case of missing follow-up to assess the patient’s health status.

### Outcomes

The primary outcome was symptomatic VTE recurrence up to 5 years of follow-up after anticoagulation discontinuation. Recurrent VTE was defined by (i) a symptomatic non-fatal recurrent PE, or (ii) a symptomatic recurrent DVT, or (iii) a fatal recurrent PE. The diagnosis of recurrent DVT was confirmed using real B mode leg ultrasound in case of initial DVT extension, contralateral recurrence of DVT, or ipsilateral DVT recurrence. The diagnosis of recurrent PE was confirmed by (i) a segmental or a more proximal thrombus on CTPA, (ii) the presence of at least one new perfusion defect of at least 75% in contrast with normal ventilation, (iii) a clinical suspicion of PE associated with a recurrent proximal DVT. The secondary outcomes were overall mortality and the phenotype of recurrent VTE (i.e., PE versus DVT). Physicians not involved in the patient’s medical care adjudicated all initial VTE events, recurrences, and deaths.

### Statistical analysis

Continuous variables were expressed as mean (standard deviation [SD]) and median (interquartile range [IQR]); categorical variables were expressed as numbers and percentages. The student t-test was used to compare means between groups in case of normal distribution and the Mann-Whitney test in non-normal distribution. A Chi-square test or exact Fisher test was used to compare proportions as appropriate.

The rates of recurrent VTE were estimated using the Kaplan Meier method. An univariable Cox proportional hazard model analysis was performed to identify risk factors of recurrent VTE and death. Hazard ratio (HR) with 95% confident interval (CI) were provided. Multivariable analyses were performed using cause specific Cox models in two stages for the risk of recurrent VTE as well as for the risk of death (competitive risk) in association with OSA. The first multivariable model was constructed by including variables whose distribution was statistically different (*p* < 0.05) between the two groups. The second multivariable model was constructed by including (i) variables associated with increased risk of recurrence or death in univariable analysis, with *p*-value < 0.15 and a frequency > 3%; and (ii) variables whose distribution was significantly different between the two groups (*p* < 0.05). In multivariable models, risk factors were considered independent for a *p*-value < 0.05. All tests were two-sided. Statistical analyses were performed using R software Version 1.0.153 –© 2009–2017 RStudio, Inc.

### Ethics statement

This study was conducted following the amended Declaration of Helsinki. The Ethics Committee of Brest University Hospital approved the study protocol (IRB approval number: CCP Ouest 6–390). Written informed consent was obtained from all participants before inclusion.

## Results

**Fig. 1:**
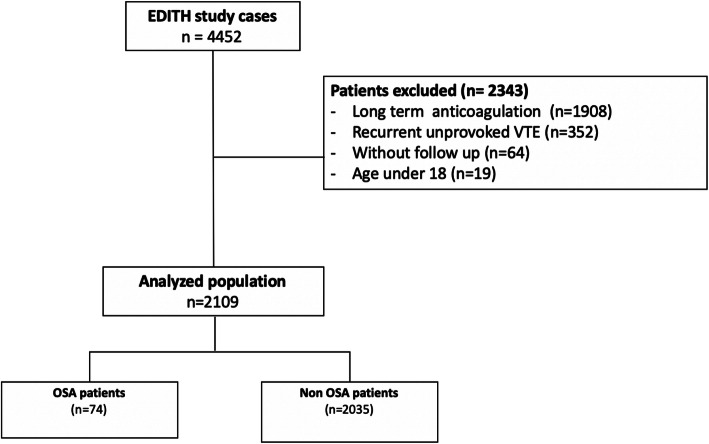
Flow chart. OSA, Obstructive Sleep Apnea; VTE, Venous Thromboembolism

Between May 2000 and June 2019, 4452 patients with objectively confirmed VTE were enrolled and prospectively followed-up. Among them, 2343 patients were excluded for the following reasons: long term anticoagulation (*n* = 1908), a recurrent VTE (*n* = 352), age under 18 years old (n = 19), and without follow up (*n* = 64) (Fig. 1). A total of 2109 patients (932 men, 1177 women) were included in the analysis.

### Baseline characteristics

Overall, 74 patients had OSA objectively confirmed by the home sleep test of polysomnography. Patients with OSA were older, predominantly obese or overweight, with history of cardiovascular and pulmonary disease. Sixty-three of them (85%) had a dedicated device (NIV or CPAP) for OSA. VTE was generally unprovoked, and clinical presentation was more often PE rather than DVT in OSA patients. Concomitant treatment (i.e., statin, antiplatelet agents) and anticoagulant therapy length were not significantly different between the two groups. The mean (standard deviation SD) follow-up of the overall population was 5.6 (4.2) years, 5.6 (4.2) years in the non-OSA group, and 5.4 (4.2) years in the OSA group (*p* = 0.70). Demographic and clinical characteristics at inclusion are presented in Table 1.

**Table 1:** Baseline characteristics

Variables		Total	Non-OSA patients	OSA patients	***p***-value
**Age (years)**	Mean (SD)	56.75 (19.4)	56.53 (19.6)	62.67 (13.3)	0.008
**Age (years)**	n (%)				0.002
	≤ 50	805 (38.2)	790 (38.8)	15 (20.3)	
	50–65	464 (22.0)	439 (21.6)	25 (33.8)	
	> 65	839 (39.8)	805 (39.6)	34 (45.9)	
**Gender**	Female n (%)	1177 (55.8)	1151 (56.6)	26 (35.1)	< 0.001
**BMI (kg/m**^**2**^**)**	Mean (SD)	26.27 (5.0)	26.10 (4.9)	30.90 (5.5)	< 0.001
**BMI (kg/m**^**2**^**)**	n (%)				< 0.001
	≤ 25	917 (44.4)	908 (45.6)	9 (12.5)	
	> 25 - ≤ 30	763 (36.9)	735 (36.9)	28 (38.9)	
	> 30 - ≤ 35	268 (13.0)	252 (12.6)	16 (22.2)	
	> 35	117 (5.7)	98 (4.9)	19 (26.4)	
**Smoking**	n (%)	973 (47.0)	936 (46.9)	37 (51.4)	0.53
**Atrial fibrillation**	n (%)	58 (2.8)	55 (2.8)	3 (4.2)	0.71
**Chronic heart failure: n (%):**	n (%)	134 (6.4)	125 (6.1)	9 (12.2)	0.07
**Kidney failure**	n (%)	83 (3.9)	73 (3.6)	10 (13.5)	< 0.001
**Stroke**	n (%)	68 (3.2)	55 (2.7)	13 (17.6)	< 0.001
**Characteristics of index VTE**	n (%)				0.032
	Isolated PE	558 (26.5)	530 (26.1)	28 (37.8)	
	PE associated with DVT	610 (29.0)	587 (28.9)	23 (31.1)	
	Isolated DVT	934 (44.4)	911 (44.9)	23 (31.1)	
**Characteristics of recurrent VTE**	n (%)				0.23
	Isolated PE	63 (25.0)	61 (25.1)	2 (22.2)	
	PE associated with proximal DVT	44 (17.5)	40 (16.5)	4 (44.4)	
	PE associated with distal DVT	20 (7.9)	19 (7.8)	1 (11.1)	
	Isolated proximal DVT	84 (33.3)	83 (34.2)	1 (11.1)	
	Isolated distal DVT	41 (16.3)	40 (16.5)	1 (11.1)	
**Anticoagulant duration**	n (%)				0.37
	≤ 90 days	190 (9.0)	184 (9.1)	6 (8.1)	
	> 90 - ≤ 180 days	543 (25.8)	527 (25.9)	16 (21.6)	
	> 180 - ≤ 360 days	895 (42.5)	866 (42.6)	29 (39.2)	
	> 360 days	478 (22.7)	455 (22.4)	23 (31.1)	
**Unprovoked VTE**	n (%)	1199 (56.9)	1148 (56.4)	51 (68.9)	0.044
**Provoked VTE**	n (%)	910 (43.1)	887 (43.6)	23 (31.1)	
**Aspirin**	n (%)	215 (10.2)	203 (10.0)	12 (16.2)	0.12
**Statin**	n (%)	192 (9.1)	181 (8.9)	11 (14.9)	0.12
**CPAP**	n (%)	63 (69.2)	0 (0.0)	63 (85.1)	< 0.001
**OSA history**	n (%)	46 (2.2)	0 (0.0)	46 (2.2)	
**OSA occurrence**	n (%)	28 (1.3)	0 (0.0)	28 (1.3)	
**PSG or HST n (%)**			17 (0.8)	57 (77.0)	
	Mean (SD)				
	Total number of apneas	88.50 (105.4)	15.64 (22.6)	114.00 (111.2)	0.002
	Number of apneas by hours	11.92 (14.4)	1.81 (2.9)	15.37 (15.2)	0.002
	Number of obstructive apneas	59.58 (80.8)	6.93 (11.1)	78.97 (86.7)	0.003
	Number of central apneas	6.40 (17.5)	1.93 (4.9)	8.05 (20.1)	0.27
	Number of mixed apneas	8.92 (19.8)	1.21 (2.2)	11.76 (22.6)	0.09
	Apnea hypopnea index by hour	31.82 (20.8)	8.15 (4.3)	38.88 (18.4)	< 0.001
**Nocturnal Desaturation**			17 (0.8)	57 (77.0)	
	Mean (SD)				
	Percentage of time < 90%	29.73 (31.3)	21.41 (32.4)	32.51 (30.7)	0.24
	Percentage of time < 85%	11.26 (22.1)	8.71 (23.0)	12.08 (22.0)	0.63
	Percentage of time < 80%	3.35 (12.1)	0.08 (0.3)	4.42 (13.8)	0.27

*BMI* body mass index, *CPAP* continuous positive airway pressure, *OSA* Obstructive Sleep Apnea, *PSG* polysomnography, *HST* Home Sleep Tests, *VTE* Venous Thromboembolism

### Risk of recurrent VTE

The 5-year cumulative incidence rates of recurrent VTE were not statistically different (*p* = 0.38) between patients with: (i) OSA history (19.2% (95% CI, 3.9–32.1); (ii) OSA occurrence (4.3% (95% CI, 0.0–12.3)); (iii) and patients without OSA (12.4% (95% CI, 10.8–13.9)).

In univariable analysis, OSA diagnosed prior index VTE (HR 1.46 (95% CI, 0.69–3.09); *p* = 0.33), OSA diagnosed after index VTE (HR 2.78 (95% CI, 0.68–11.32); *p* = 0.328), AHI and nocturnal desaturation were not associated with an increased risk of recurrence (Table 2).

**Table 2:** Risk factor for recurrent VTE

Variables	Univariable analysis HR, 95% CI	***p***-value	Multivariable analysis 1 HR, 95% CI	***p***-value	Multivariable analysis 2 HR, 95% CI	***p***-value
**Age range (years)**						
≤ 50	Ref.		Ref.		Ref.	
]50–65]	1.9 (1.3–2.7)	< 0.001	1.6 (1.1–2.3)	0.020	1.6 (1.1–2.3)	0.019
> 65	2.4 (1.8–3.3)	< 0.001	2.1 (1.5–3.0)	< 0.001	2.2 (1.5–3.0)	< 0.001
**BMI range (kg/m**^**2**^**)**						
≤ 25	Ref.		Ref.		Ref.	
]25–30]	1.1 (0.8–1.5)	0.48	0.9 (0.7–1.2)	0.58	0.9 (0.7–1.3)	0.69
]30–35]	1.1 (0.7–1.6)	0.70	1.0 (0.7–1.5)	0.90	1.0 (0.7–1.5)	0.83
> 35	0.6 (0.3–1.3)	0.21	0.7 (0.4–1.4)	0.31	0.7 (0.4–1.4)	0.35
**Females**	0.7 (0.6–0.9)	0.006	0.8 (0.6–1.1)	0.10	0.8 (0.6–1.0)	0.09
**Chronic cardiac failure history**	0.6 (0.3–1.2)	0.13	0.4 (0.2–0.9)	0.018	0.4 (0.2–0.8)	0.007
**Cerebral stroke**	0.8 (0.4–1.8)	0.58	0.6 (0.3–1.5)	0.31		
**Statins**	1.1 (0.7–1.7)	0.69	1.0 (0.7–1.6)	0.93		
**Antiplatelet agents**	1.0 (0.6–1.5)	0.82	0.9 (0.5–1.4)	0.51		
**Smoking**	0.9 (0.7–1.2)	0.54				
**COPD**	1.4 (0.9–2.2)	0.19	1.0 (0.6–1.7)	0.97		
**Cancer**	1.8 (1.2–2.8)	0.005	2.0 (1.2–3.4)	0.008	2.0 (1.2–3.4)	0.009
**Familial history of VTE**	1.1 (0.8–1.5)	0.47				
**Unprovoked VTE**	1.7 (1.3–2.2)	< 0.001	2.1 (1.4–3.1)	0.001	2.0 (1.3–3.0)	0.001
**OSA history**	1.5 (0.7–3.0)	0.33	1.6 (0.7–3.4)	0.26	1.4 (0.6–2.9)	0.42
**OSA occurrence**	2.8 (0.7–11.3)	0.15	2.4 (0.6–9.8)	0.23	2.3 (0.6–9.6)	0.24
**AHI**	1.0082 (0.9775–1.040)	0.61				
**AHI (continuous range of 10)**	1.1 (0.8–1.5)	0.72				
**Nocturnal desaturation (hour index)**	0.9776 (0.9080–1.052)	0.97				
**Anticoagulation duration**	0.9995 (0.9989–0.9999)	0.54	0.9974 (0.9989–1.0005)	0.52	0.9998 (0.999–1.0006)	0.56

*BMI* body mass index, *OSA* Obstructive Sleep Apnea, *AHI* apnea hypopnea index, *VTE* Venous Thromboembolism Multivariable model 1 included variables associated with an increased risk of recurrence in univariable analysis and multivariable model 2 included baseline characteristics distributed differently between OSA and non-OSA patients

In multivariable model 1, which included variables associated with an increased risk of recurrence in univariable analysis, the risk of recurrent VTE was not significantly increased in patients with OSA history (HR 1.37 (95% CI, 0.64–2.94); *p* = 0.46) or OSA occurrence (HR 2.34 (95% CI, 0.57–9.62); *p* = 0.24) as compared to other patients (Table 2). In multivariable model 2, which included baseline characteristics distributed differently between OSA and non-OSA patients, and variables associated with an increased risk of recurrence in univariable analysis, the predictors of recurrent VTE were similar to those found in the first multivariable model (Table 2). The main risk factors of recurrence were older age, unprovoked VTE, and cancer. Similar results were observed in sensitivity analysis on the risk of recurrent VTE in patients with pooled diagnosis of OSA (eTable 1).

### Index and recurrent VTE clinical presentation

Recurrent VTE occurred in 252 patients during the follow-up after anticoagulation discontinuation; nine of these recurrences occurred in OSA patients and 243 in non-OSA patients (Table 3). Among the 252 recurrences of VTE, 125 (49,6%) were isolated DVT, 63 (25%) were isolated PE, 64 (25,4%) were PE with DVT (Table 3).

**Table 3:** Phenotype of VTE

Index VTE n (%)	OSA-	OSA+	***p***-value
**as PE with or without DVT**	1117 (54.9)	51 (68.9)	0.023
**as isolated DVT**	918 (45.1)	23 (31.1)	
**Recurrent VTE n (%)**			0.09
**as PE with or without DVT**	120 (49.4)	7 (77.8)	
**as isolated DVT**	123 (50.6)	2 (22.2)	

*OSA* Obstructive Sleep Apnea, *VTE* Venous Thromboembolism, *DVT* deep vein thrombosis, *PE* pulmonary embolism

The clinical presentation of index VTE and recurrent VTE were statistically different: 1117 (55%) patients without OSA and 51 (68.9%) patients with OSA had an index PE with or without DVT (*p* = 0.023), 120 (49.4%) patients without OSA and seven patients (77.8%) patients with OSA had a recurrent VTE as PE with or without DVT (*p* = 0.09).

### Mortality

During follow-up, death from any cause occurred in 155 (7.3%) patients: 2 of the 74 OSA patients (2.7%) and 153 of 2035 non-OSA patients (7.5%) (*p* = 0.295). In univariable analysis, there was no increased risk of death in patients with a history of OSA compared to non-OSA patients (HR 0.75 (95% CI, 0.19–3.02); *p* = 0.70) (eTable 2). AHI was not associated with an increased risk of death as well as nocturnal desaturation. After adjustment on variable associated with mortality in univariable analysis, the risk of death was not greater in OSA history patients as compared to other patients (HR 0.18 (95% CI, 0.02–1.31); *p* = 0.09) (eTable 2).

## Discussion

In the present study, including 2109 patients with acute symptomatic VTE, followed-up after anticoagulation discontinuation, the presence of OSA diagnosed before or after index VTE, treated by CPAP in the majority of cases (85%) was not significantly associated with an increased risk of VTE recurrence, nor with increased overall mortality, as well as AHI and nocturnal desaturation.

Although OSA could be considered as a chronic syndrome with exposition to intermittent hypoxemia over the years before treatment and diagnosis, we considered two groups of patients, one with a history of OSA before the index VTE and one with an occurrence of OSA after the index VTE to evaluate its effect over the time. We found that OSA’s occurrence tended to increase the recurrence risk compared to OSA’s history. The effect of OSA pooled, as one variable was similar.

Alonso-Fernández et al. evaluated the association between OSA and the risk of recurrent PE after anticoagulation discontinuation in 120 patients with a first episode of PE [28]. OSA was identified after index VTE in 71 patients. OSA was associated with an increased risk of VTE recurrence (HR 4.05 (95% CI, 1.18–13.91) *p* = 0.026). Firstly, OSA was diagnosed with HST after PE, while in the present study, most OSA diagnoses were made before index VTE. However, there was no difference statistically significant in the risk of recurrent VTE between OSA diagnosed before and after index VTE. Secondly, AHI ≥ 10/h was identified as an independent risk factor, but AHI ≥ 30/h was not. In the present study, OSA diagnosis was mainly severe OSA: the mean (SD) AHI (h-1) was higher (38.88 ± 18.4 vs 21.1 ± 20.5), the mean (SD) desaturation index (h-1) was higher (41.62 ± 35.15 vs 18.3 ± 19.7), and CPAP treatment was more frequent in OSA patients (63 of 74 (85.1%) vs 31 of 71 (43.7%)). The present study was not powered enough to evaluate CPAP effect on VTE recurrence risk but may have led to reducing the risk of recurrence as the majority of patients were treated. Some experimental studies suggested an endothelial dysfunction improvement with this treatment in OSA patients [34, 35]. Xie et al. [27], in a prospective study including 97 patients with 32 OSA with PE anticoagulated with warfarin during 6 months, found a higher incidence of PE recurrence after anticoagulation discontinuation in OSA patients (21.43% vs 6.78%; *p* = 0.047). Nevertheless, recurrent VTE risk factors were not collected, and OSA was less severe, with only 12.5% of OSA patients with CPAP.

The results of the present study are consistent with the “RIETE registry”: pre-existing OSA was not associated with recurrent VTE [29]. The study had several limitations: RIETE does not include information related to use of CPAP, AHI, and the relatively short follow up limited the conclusion regarding the association between long term VTE recurrence and OSA.

The clinical presentation of index VTE was significantly different between groups; there was more index PE with or without DVT in OSA patients. Recurrence as PE with or without DVT tends to be more frequent in OSA patients without reaching statistical significance. In the general population, the proportion of PE with or without DVT and DVT alone are similar [3], and DVT are more frequent as an index or recurrent VTE in unprovoked VTE [4]. A predominance of PE with or without DVT over DVT alone has also been found in chronic obstructive pulmonary disease patients suggesting an influence of respiratory disease on VTE clinical presentation [30]. The higher proportion of PE has been related to poor outcomes in the OSA population [36]; however, it was not associated with an increased risk of OSA patients’ mortality compared to non-OSA patients.

Consistent with previous studies evaluating the risk of VTE recurrence, unprovoked VTE, older age, cancer, and male gender were associated with a higher risk of VTE recurrence [4, 37–39].

The strengths of our study are related to (i) the prospective patient’s recruitment with documented VTE as PE, with or without DVT, or isolated DVT, that were followed-up for an extended period with a median up to 4.8 (2.5–8.0) years, with scheduled clinical reevaluation every 6 or 12 months, (ii) OSA assessment in all patients based on HST or polysomnography results including AHI, nocturnal desaturation and the characteristics of apneas, (iii) the use of predefined, validated and objective criteria for all cases of recurrent VTE and predefined criteria for assessing the cause of deaths, which were adjudicated by physicians who were not involved in patient care, (iv) the multicenter design.

Several limitations should be considered. Obesity can be a confounding factor associated with VTE and OSA and might interact with its effect on recurrence. This is an observational study with data collected during a considerable period, with a random occurrence of OSA diagnosis and different sleep monitoring devices over the years. Moreover, many OSA patients included had CPAP treatment without random assignation, limiting additional evaluation of its influence and evaluating severe OSA on VTE recurrence. Sixty-four patients with OSA were excluded because on indefinite anticoagulation, the number of patients with OSA included in this study was therefore restricted. Lastly, only patients referred to the cohort centers were included in the study, they might have had more comorbidities and an index VTE with more severe clinical features than the general population with VTE. They also might have had a better prognosis due to a follow up by experienced teams with expertise in VTE, and we cannot exclude that clinical presentation have been influenced by ambulatory care of DVT.

## Conclusion

In this study, we failed to find an association between the risk of recurrent VTE, death and moderate to severe OSA after anticoagulation discontinuation. Nevertheless, larger studies are needed to evaluate OSA’s effect on VTE recurrence, given the previous evidence suggesting an association between OSA and the risk of recurrent VTE and considering the pro-coagulant state induced by intermittent hypoxemia. The effectiveness of CPAP and/or extended anticoagulation remains unknown and call for additional studies in patients with OSA and VTE.

## Supplementary Information

**Additional file 1: eTable 1.** Risk factor for recurrent VTE with OSA pooled.

**Additional file 2: eTable 2.** Risk factors associated with mortality.

**Additional file 3: eTable 3.** Risk factors associated with mortality with OSA pooled.

## Data Availability

The datasets used and/or analyzed during the current study are available from the corresponding author on reasonable request.
