# Supplementary material for: Association between obstructive sleep apnea and venous thromboembolism recurrence: results from a French cohort
Source: Thromb J. 2022 Jan 4;20:1. doi: 10.1186/s12959-021-00358-8 (PMC8725561; doi:10.1186/s12959-021-00358-8)
Supplement: Supplementary file 1 — Additional file 1: eTable 1. Risk factor for recurrent VTE with OSA pooled. [file 12959_2021_358_MOESM1_ESM.docx]

**eTable 1. Risk factor for recurrent VTE with OSA pooled**

| **Variables** | | **Univariable analysis**  **HR, 95% CI** | **p-value** | **Multivariable analysis**  **HR, 95% CI** | **p-value** |
| --- | --- | --- | --- | --- | --- |
| **Age range (years)** | |  | | | |
|  | ≤50 | Ref. |  | Ref. |  |
|  | ]50-65] | 1.9 (1.3-2.7) | <0.001 | 1.6 (1.1-2.3) | 0.016 |
|  | > 65 | 2.4 (1.8-3.3) | <0.001 | 2.2 (1.5-3.0) | <0.001 |
| **BMI range (kg/m²)** | |  | | | |
|  | ≤25 | Ref. |  | Ref. |  |
|  | ]25-30] | 1.1 (0.8-1.5) | 0.48 | 0.9 (0.7-1.2) | 0.60 |
|  | ]30-35] | 1.1 (0.7-1.6) | 0.70 | 1.0 (0.7-1.5) | 0.85 |
|  | > 35 | 0.6 (0.3-1.3) | 0.21 | 0.7 (0.4-1.5) | 0.43 |
| **Females** | | 0.7 (0.6-0.9) | 0.006 | 0.8 (0.6-1.0) | 0.09 |
| **Chronic cardiac failure history** | | 0.6 (0.3-1.2) | 0.13 | 0.4 (0.2-0.9) | 0.02 |
| **Cerebral stroke** | | 0.8 (0.4-1.8) | 0.58 | 0.7 (0.3-1.7) | 0.42 |
| **Statins** | | 1.1 (0.7-1.7) | 0.69 | 1.0 (0.7-1.6) | 0.94 |
| **Antiplatelet agents** | | 1.0 (0.6-1.5) | 0.82 | 0.8 (0.5-1.4) | 0.49 |
| **Smoking** | | 0.9 (0.7-1.2) | 0.54 |  |  |
| **COPD** | | 1.4 (0.9-2.2) | 0.19 | 1.0 (0.6-1.7) | 0.93 |
| **Cancer** | | 1.8 (1.2-2.8) | 0.005 | 2.0 (1.2-3.4) | 0.008 |
| **Familial history of VTE** | | 1.1 (0.8-1.5) | 0.47 |  |  |
| **Unprovoked VTE** | | 1.7 (1.3-2.2) | <0.001 | 2.1 (1.4-3.1) | 0.001 |
| **OSA pooled** | | 1.0 (0.5-2.0) | 0.95 | 1.0 (0.5-2.0) | 0.97 |
| **AHI** | | 1.0 (1.0-1.0) | 0.61 |  |  |
| **AHI (continuous range of 10)** | | 1.1 (0.8-1.5) | 0.72 |  |  |
| **Nocturnal desaturation (hour index)** | | 1.0 (1.0-1.0) | 0.97 |  |  |
| **Anticoagulation duration** | | 1.0 (1.0-1.0) | 0.054 | 1.0 (1.0-1.0) | 0.08 |

BMI, body mass index; OSA, Obstructive Sleep Apnea; AHI, apnea hypopnea index; VTE, Venous Thromboembolism; COPD, chronic obstructive pulmonary disease
