# Supplementary material for: Association between obstructive sleep apnea and venous thromboembolism recurrence: results from a French cohort
Source: Thromb J. 2022 Jan 4;20:1. doi: 10.1186/s12959-021-00358-8 (PMC8725561; doi:10.1186/s12959-021-00358-8)
Supplement: Supplementary file 2 — Additional file 2: eTable 2. Risk factors associated with mortality. [file 12959_2021_358_MOESM2_ESM.pdf]

eTable 2. Risk factors associated with mortality

Variables		Univariable analysis HR, 95% CI	p-value	Multivariable analysis HR, 95% CI	p-value
**Age (years)**		1.094 (1.078-1.110)	<0.001		
**Age range (years)**					
	≤50	Ref.		Ref.	
	]50-65]	6.9 (2.8-17.1)	<0.001	4.5 (1.8-11.8)	0.002
	> 65	27.3 (12.0-61.9)	<0.001	14.1 (6.0-33.4)	<0.001
**BMI**		1.0 (0.9-0.99)	0.015		
**BMI range (kg/m²)**					
	≤25	Ref.		Ref.	
	]25-30]	0.9 (0.6-1.3)	0.49	0.8 (0.5-1.1)	0.16
	]30-35]	0.7 (0.4-1.2)	0.16	0.6 (0.3-1.1)	0.08
	> 35	0.4 (0.2-1.1)	0.08	0.8 (0.3-2.4)	0.75
**Females**		0.8 (0.6-1.1)	0.20	1.0 (0.7-1.4)	0.78
**Smoking**		1.2 (0.9-1.7)	0.21		
**Cerebral ischemic attack**		3.1 (1.7-5.4)	<0.001	1.7 (0.9-3.4)	0.12
**Arteriopathy**		4.6 (2.7-8.0)	<0.001	2.1 (1.1-4.2)	0.025
**Atrial fibrillation**		8.6 (5.5-13.5)	<0.001	1.5 (0.8-2.6)	0.19
**Transient cerebral ischemic attack**		2.1 (0.7-6.7)	0.20		
**Familial History of VTE**		0.3 (0.2-0.6)	<0.001	0.6 (0.3-0.9)	0.028
**Statins**		0.5 (0.3-1.1)	0.10	0.2 (0.1-0.5)	<0.001
**Antiplatelet agents**		3.1 (2.1-4.6)	<0.001	1.1 (0.7-1.9)	0.67
**Restrictive ventilation disorder**		4.0 (1.6-9.8)	0.002	3.6 (1.3-9.6)	0.012
**Bronchiectasis**		0.7 (0.1-4.6)	0.66		
**Asthma**		1.7 (0.4-6.9)	0.45		
**COPD**		3.3 (2.1-5.1)	<0.001	1.4 (0.9-2.4)	0.17
**Acute cardiac failure history**		11.0 (6.6-18.3)	<0.001	3.9 (2.1-7.4)	<0.001
**Chronic cardiac failure history**		5.0 (3.4-7.3)	<0.001	1.8 (1.1-3.0)	0.025
**Cancer**		5.4 (3.6-8.0)	<0.001	5.7 (3.1-10.5)	<0.001
**Unprovoked VTE**		1.6 (1.2-2.3)	0.004	1.8 (1.1-3.0)	0.024
**OSA history**		0.8 (0.2-3.0)	0.69	0.2 (0.0-1.3)	0.90
**OSA occurrence**		0.0 (0.0-Inf)	0.99	0.0 (0.0-Inf)	0.99
**Anticoagulation duration range (days)**					
	90-180	0.4 (0.2-0.6)	<0.001	0.6 (0.3-0.9)	0.028
	180-360	0.4 (0.3-0.6)	<0.001	0.5 (0.3-0.8)	0.002
	> 360	0.4 (0.3-0.7)	0.002	0.5 (0.3-0.8)	0.01
**Anticoagulation duration**		1.0 (1.0-1.0)	0.40		

BMI, body mass index; OSA, Obstructive Sleep Apnea; VTE, Venous Thromboembolism; COPD, chronic obstructive pulmonary disease
