# Supplementary material for: Association between obstructive sleep apnea and venous thromboembolism recurrence: results from a French cohort
Source: Thromb J. 2022 Jan 4;20:1. doi: 10.1186/s12959-021-00358-8 (PMC8725561; doi:10.1186/s12959-021-00358-8)
Supplement: Supplementary file 3 — Additional file 3: eTable 3. Risk factors associated with mortality with OSA pooled. [file 12959_2021_358_MOESM3_ESM.docx]

**eTable 3. Risk factors associated with mortality with OSA pooled**

| **Variables** | | **Univariable analysis**  **HR, 95% CI** | **p-value** | **Multivariable analysis**  **HR, 95% CI** | **p-value** |
| --- | --- | --- | --- | --- | --- |
| **Age (years)** | | 1.094 (1.078-1.110) | <0.001 |  |  |
| **Age range (years)** | |  | | | |
|  | ≤50 | Ref. |  | Ref. |  |
|  | ]50-65] | 6.9 (2.8-17.1) | <0.001 | 4.5 (1.8-11.7) | 0.002 |
|  | > 65 | 27.3 (12.0-61.9) | <0.001 | 14.1 (6.0-33.3) | <0.001 |
| **BMI** | | 1.0 (0.9-0.99) | 0.015 |  |  |
| **BMI range (kg/m²)** | |  | | | |
|  | ≤25 | Ref. |  | Ref. |  |
|  | ]25-30] | 0.9 (0.6-1.3) | 0.49 | 0.8 (0.5-1.1) | 0.18 |
|  | ]30-35] | 0.7 (0.4-1.2) | 0.16 | 0.6 (0.3-1.1) | 0.09 |
|  | > 35 | 0.4 (0.2-1.1) | 0.08 | 0.9 (0.3-2.5) | 0.83 |
| **Females** | | 0.8 (0.6-1.1) | 0.20 | 0.9 (0.7-1.4) | 0.74 |
| **Smoking** | | 1.2 (0.9-1.7) | 0.21 |  |  |
| **Cerebral ischemic attack** | | 3.1 (1.7-5.4) | <0.001 | 1.8 (0.9-3.4) | 0.11 |
| **Arteriopathy** | | 4.6 (2.7-8.0) | <0.001 | 2.1 (1.1-4.1) | 0.03 |
| **Atrial fibrillation** | | 8.6 (5.5-13.5) | <0.001 | 1.5 (0.8-2.6) | 0.20 |
| **Transient cerebral ischemic attack** | | 2.1 (0.7-6.7) | 0.20 |  |  |
| **Familial History of VTE** | | 0.3 (0.2-0.6) | <0.001 | 0.6 (0.3-0.9) | 0.028 |
| **Statins** | | 0.5 (0.3-1.1) | 0.10 | 0.2 (0.1-0.5) | <0.001 |
| **Antiplatelet agents** | | 3.1 (2.1-4.6) | <0.001 | 1.1 (0.7-1.9) | 0.65 |
| **Restrictive ventilation disorder** | | 4.0 (1.6-9.8) | 0.002 | 3.5 (1.3-9.4 | 0.013 |
| **Bronchiectasis** | | 0.7 (0.1-4.6) | 0.66 |  |  |
| **Asthma** | | 1.7 (0.4-6.9) | 0.45 |  |  |
| **COPD** | | 3.3 (2.1-5.1) | <0.001 | 1.5 (0.9-2.5) | 0.15 |
| **Acute cardiac failure history** | | 11.0 (6.6-18.3) | <0.001 | 3.9 (2.1-7.4) | <0.001 |
| **Chronic cardiac failure history** | | 5.0 (3.4-7.3) | <0.001 | 1.8 (1.1-2.9) | 0.028 |
| **Cancer** | | 5.4 (3.6-8.0) | <0.001 | 5.6 (3.1-10.4 | <0.001 |
| **Unprovoked VTE** | | 1.6 (1.2-2.3) | 0.004 | 1.8 (1.1-3.0) | 0.024 |
| **OSA pooled** | | 0.4 (0.1-1.5) | 0.16 | 0.1 (0.0-0.9) | 0.042 |
| **Anticoagulation duration range (days)** | |  | | | |
|  | 90-180 | 0.4 (0.2-0.6) | <0.001 | 0.6 (0.3-0.9) | 0.029 |
|  | 180-360 | 0.4 (0.3-0.6) | <0.001 | 0.5 (0.3-0.8) | 0.002 |
|  | > 360 | 0.4 (0.3-0.7) | 0.002 | 0.5 (0.3-0.8) | 0.010 |
| **Anticoagulation duration** | | 1.0 (1.0-1.0) | 0.40 |  |  |

BMI, body mass index; OSA, Obstructive Sleep Apnea; VTE, Venous Thromboembolism; COPD, chronic obstructive pulmonary disease
